# Glycosuria medicated with ipragliflozin and nifedipine or ipragliflozin and candesartan: a case report

**DOI:** 10.1186/1752-1947-8-428

**Published:** 2014-12-16

**Authors:** Shuichi Okada, Ryo Shibusawa, Yuko Tagaya, Tsugumichi Saito, Eijiro Yamada, Yoko Shimoda, Tetsurou Satoh, Junichi Okada, Masanobu Yamada

**Affiliations:** Department of Medicine and Molecular Science, Gunma University Graduate School of Medicine, 3-39-15 Showa-machi, Maebashi, Gunma 371-8511 Japan

**Keywords:** Candesartan, Glycosuria, Ipragliflozin, Nifedipine

## Abstract

**Introduction:**

Animal studies have reported that treatment with angiotensin II receptor blockers reduced kidney sodium-dependent glucose cotransporter expression. We therefore hypothesized that patients with hypertension treated with an angiotensin II receptor blocker (candesartan) would probably have an increased response to sodium-dependent glucose cotransporter inhibitor therapy (ipragliflozin) compared with patients treated with alternative hypertensive medications such as calcium channel blockers (nifedipine).

Although sodium-dependent glucose cotransporter inhibitor (ipragliflozin) is a new anti-diabetic medicine, the clinical efficacy in the Japanese population has not been fully evaluated. We compared the combined effect of angiotensin II receptor blocker candesartan plus ipragliflozin with nifedipine plus ipragliflozin therapy and found that the combination of candesartan plus ipragliflozin was more effective in increasing glycosuria and lowering plasma glucose.

**Case presentation:**

A 57-year-old Japanese man with essential hypertension was treated with candesartan. Candesartan was switched to nifedipine for the initial 10 days of an observation period and 5 days later he was started on ipragliflozin (day 6 of nifedipine treatment) with nifedipine for the next 5 days. Thereafter (from day 11 to day 20), candesartan was started instead of nifedipine and ipragliflozin was continued. In the last 5 days ipragliflozin was stopped and he was treated with candesartan alone. Neither nifedipine alone (0.038+/-0.004) nor candesartan alone (0.048+/-0.006) produce any trace amount of glycosuria. However, the extent of glycosuria under ipragliflozin with candesartan treatment (37.5+/-8.45) was significantly greater than that of ipragliflozin with nifedipine (23.75+/-0.35; *P*<0.05).

**Conclusion:**

Candesartan demonstrated additive actions with ipragliflozin to increase glycosuria compared to ipragliflozin with nifedipine treatment.

## Introduction

Sodium-dependent glucose cotransporter 2 (SGLT2) inhibitors are a new class of anti-diabetic agents and are currently attracting clinicians’ attention in our country, Japan [1]. However, there is a scarcity of information on the effectiveness of SGLT2 inhibitors in the Japanese population. Angiotensin II receptor blockers (ARBs) reduce aldosterone secretion by mainly antagonizing the angiotensin I receptor but are less likely to regulate glucose reabsorption in the kidney [2]. Nevertheless, three case reports have indicated that angiotensin-converting enzyme (ACE) inhibitor resulted in glycosuria without any other side effects including kidney dysfunction [3]. Thus, we hypothesized that ACE inhibition may increase glycosuria through actions on the angiotensin II receptor and reduction of SGLT2 levels in hypertensive patients. To compare directly the effect of the combination of ipragliflozin and nifedipine with the combination of ipragliflozin and candesartan, we treated the same hypertensive Japanese man with these combination therapies and observed a further increase in glycosuria with the combination therapy of ipragliflozin and candesartan. This is the first case report to show the direct comparison of the combination therapy of ipragliflozin and candesartan and the combination of ipragliflozin and nifedipine on glycosuria in the same hypertensive man.

## Case presentation

**Figure 1:**
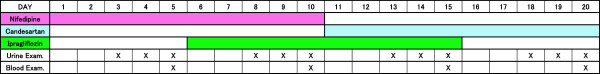
**Schedule of drug administration and sample collection.** Briefly, candesartan (8mg/day) was switched to nifedipine (10mg/day) for the initial 10 days of the observation period (indicated by pink color bar) and 5 days later he started to take ipragliflozin (50mg/day) with nifedipine after breakfast for the next 5 days (indicated by green color bar). Thereafter, candesartan (8 mg/day) was started again instead of nifedipine (indicated by blue color bar) and ipragliflozin was continued. In the last 5 days ipragliflozin was stopped (Figure 1) and the medication was candesartan alone. Abbreviations: exam., examination.

A 57-year-old Japanese man with essential hypertension diagnosed 2 years earlier was treated with candesartan (8mg/day) following the initial diagnosis. He did not have any complications including diabetes mellitus except for essential hypertension based on annual comprehensive medical check-up. After he gave informed consent, candesartan (8mg/day) was switched to nifedipine (10mg/day) for the initial 10 days of an observation period (Figure 1, indicated by pink color bar) and 5 days later he was started on ipragliflozin (day 6 of nifedipine treatment; 50mg/day) with nifedipine after breakfast for the next 5 days (Figure 1, indicated by green color bar). Thereafter (from day 11 to day 20), candesartan (8mg/day) was started instead of nifedipine (Figure 1, indicated by blue color bar) and ipragliflozin was continued. For the last 5 days of candesartan treatment the ipragliflozin was stopped (Figure 1) and he was treated with candesartan alone.

Approximately 3.5 hours after breakfast random urine and blood samples were collected as indicated in the schedule shown in Figure 1. According to the drug pharmacokinetics data, nifedipine is rapidly absorbed, reaching maximum plasma concentration within 3 hours and returns to basal level within 24 hours [4]. Therefore, we collected urine samples at days 3, 4, and 5 and a blood sample at day 5 of nifedipine treatment. Ipragliflozin is also rapidly absorbed, reaching maximum plasma concentration within 3 hours [5]. The plasma half-life of ipragliflozin is 10.0 to 13.3 hours following an initial dose of ipragliflozin [5]. Therefore, we collected urine samples at days 8, 9, and 10 and a blood sample at day 10 for the combined ipragliflozin plus nifedipine treatments. Candesartan reaches maximum plasma concentration within 6 hours with recovery to basal level within 24 hours [6]. Urine was collected at days 13, 14, and 15 and a blood sample at day 15 for the combination of ipragliflozin with candesartan treatments. We also collected urine samples at days 18, 19, and 20 and a blood sample at day 20 for candesartan treatment alone. The blood samples were immediately centrifuged at room temperature and serum was stored at -80°C. The clinical laboratory test was submitted to Special Reference Laboratories (Tokyo, Japan). Glycosuria was calculated as mean+/-standard deviation from three independent samples and statistical analysis was performed to determine statistical differences between the means with a *P* value less than 0.05 for statistical significance by InStat 2.00 program.

**Figure 2:**
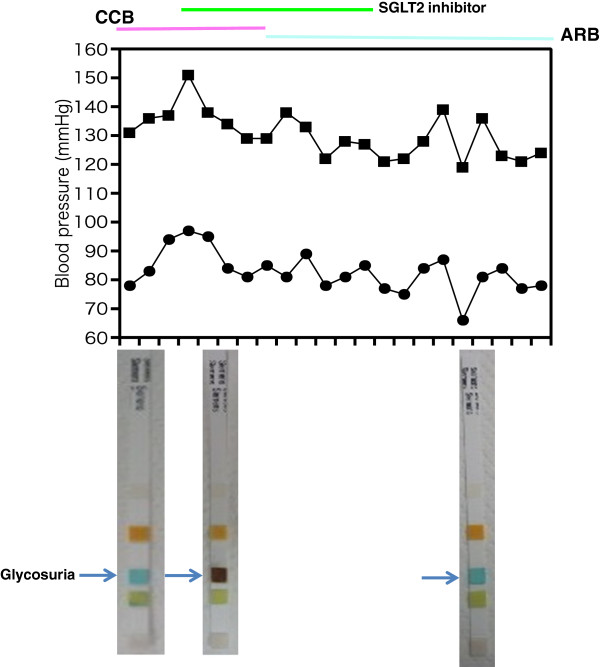
**Change of blood pressure and glycosuria.** Closed square indicates systolic pressure and closed circle indicates diastolic pressure in the upper panel. In the lower panel glycosuria is indicated by an arrow. Abbreviations: ARB, angiotensin II receptor blocker; CCB, calcium channel blocker; SGLT2, sodium-dependent glucose cotransporter 2.

As shown in Figure 2, after 3 hours (B) from the initial ipragliflozin administration (A) there was an increase in glycosuria that returned to normal basal level by 48 hours from the last ipragliflozin administration (C). However, there was no significant effect on systolic (nifedipine, calcium channel blocker, CCB, alone, 134.5+/-2.65; ipragliflozin with CCB, 133.5+/-3.7; ipragliflozin with ARB, candesartan, 123.25+/-2.63; ARB alone, 134.25+/-4.64) or diastolic blood pressure (CCB alone, 85+/-6.68; ipragliflozin with CCB, 88+/-4.97; ipragliflozin with ARB, 80+/-3.56; ARB alone, 84+/-2.45; Figure 2 and Table 1). Throughout the entire observation period we did not observe any adverse effect and there were no detectable hypotensive episodes

**Table 1:** **Blood chemistry results**

	Nifedipine	Nifedifine + Ipragliflozin	Cansdesartan + Ipragliflozin	Candesartan
BS(mg/dl)	102	105	104	101
IRI(μU/ml)	4.34	3.93	4.81	3.36
GA(%)	13.4	13.4	13.6	13.1
BUN(mg/dl)	18.8	16.5	20.8	16.7
SCr(mg/dl)	1.01	1.04	1.01	0.92
eGFR(ml/min/1.73m^2^)	60.1	58.2	60.1	66.6
Na(mEq/l)	142	143	143	139
K(mEq/l)	4	4.1	4	3.9
Cl(mEq/l)	105	107	108	103

Abbreviations: *BS* blood suger, *BUN* blood urea nitrogen, *Cl* chlorine; *eGFR* estimated glomerular filtration rate, *GA* glycoalbumin, *IRI* immunoreactive insulin, *K* potassium, *Na* sodium, *SCr* serum creatinine.

A summary of the blood examination is shown in Table 1. There were no significant changes in his blood glucose, insulin, glycoalbumin, blood urea nitrogen creatinine, estimated glomerular filtration rate (CCB alone, 60.1; ipragliflozin with CCB, 58.2; ipragliflozin with ARB, 60.1; ARB alone; 66.6), potassium, and chlorine throughout the observation period. In addition, his albuminuria index remained within the normal range through the observation period (data not shown).

**Figure 3:**
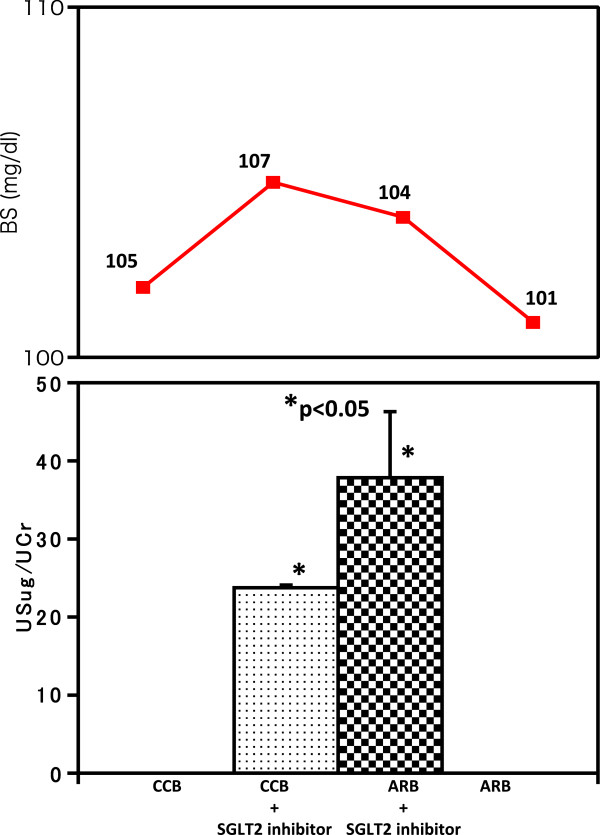
**Change of glycosuria.** In the lower panel Y axis indicates the value of glycosuria calculated as that urine sugar was divided by urine creatinine. Abbreviations: ARB, angiotensin II receptor blocker; BS, blood sugar; CCB, calcium channel blocker; SGLT2, sodium-dependent glucose cotransporter 2; UCr, urine creatinine; USug, urine sugar.

In Figure 3 we show the change in glycosuria. We confirmed that neither nifedipine alone (0.038+/-0.004) nor candesartan alone (0.048+/-0.006) produce any trace amount of glycosuria and this is consistent with glycoalbumin value indication without diabetes mellitus (Table 1 and Figure 3). However, the extent of glycosuria under ipragliflozin with candesartan treatment (37.5+/-8.45) was significantly greater than that of ipragliflozin with nifedipine (23.75+/-0.35; Figure 3, *P*<0.05).

## Discussion

This report describes a very important clinical observation that patients treated with an ARB (candesartan) will probably have an increased response to SGLT2 inhibitor therapy (ipragliflozin) compared with patients treated with alternative hypertensive medications such as CCBs (nifedipine). Although CCBs are not known to modulate SGLT2 function, ARB was recently reported to downregulate SGLT2 expression in a hypertension rat model [7]. Thus, care must be taken to carefully monitor patients treated with both ARBs and SGLT2 inhibitors to determine the extent of drug–drug interactions to increase glycosuria and dehydration particularly in the aging patient population.

This case report also indicates another important aspect of SGLT2 therapy. SGLT2 inhibition is reported to reduce the threshold of glycosuria from 180mg/dL plasma glucose to 21mg/dL plasma glucose [8]. However, here, in spite of the normal glucose tolerance there was no effect on plasma glucose (Table 1) nor did we observe any indication of a hypoglycemic episode. Thus, the compensation of the liver by increasing gluconeogenesis apparently contributed to the protection against hypoglycemia in this patient. In fact, recently dapagliflozin treatment was reported to increase endogenous glucose production [9]. Therefore the balance between insulin sensitivity and insulin resistance in the liver is very crucial to the clinical application of SGLT2 inhibitors.

## Conclusions

Based on our observation, we speculated that patients treated with an ARB (candesartan) would probably have an increased response to SGLT2 inhibitor therapy (ipragliflozin) compared with patients treated with alternative hypertensive medications such as CCBs (nifedipine). Care will be required to carefully monitor patients treated with both ARBs and SGLT2 inhibitors to determine the extent of drug–drug interactions to increase glycosuria and dehydration particularly in the aging patient population.

## Consent

Written informed consent was obtained from the patient for publication of this case report and any accompany images. A copy of the written consent is available for review by the Editor-in-Chief of this journal.

## Abbreviations

ACE: Angiotensin-converting enzyme
ARB: Angiotensin II receptor blocker
CCB: Calcium channel blocker
SGLT2: Sodium-dependent glucose cotransporter 2.
